# Matrix Profile-Based Interpretable Time Series Classifier

**DOI:** 10.3389/frai.2021.699448

**Published:** 2021-10-20

**Authors:** Riccardo Guidotti, Matteo D’Onofrio

**Affiliations:** Department of Computer Science, University of Pisa, Pisa, Italy

**Keywords:** interpretable machine learning, explainable artificial intelligence, time series classification, time-series pattern discovery, shapelet-based decision tree, transparent classifier

## Abstract

Time series classification (TSC) is a pervasive and transversal problem in various fields ranging from disease diagnosis to anomaly detection in finance. Unfortunately, the most effective models used by Artificial Intelligence (AI) systems for TSC are not interpretable and hide the logic of the decision process, making them unusable in sensitive domains. Recent research is focusing on explanation methods to pair with the obscure classifier to recover this weakness. However, a TSC approach that is transparent by design and is simultaneously efficient and effective is even more preferable. To this aim, we propose an interpretable TSC method based on the patterns, which is possible to extract from the Matrix Profile (MP) of the time series in the training set. A smart design of the classification procedure allows obtaining an efficient and effective transparent classifier modeled as a decision tree that expresses the reasons for the classification as the presence of discriminative subsequences. Quantitative and qualitative experimentation shows that the proposed method overcomes the state-of-the-art interpretable approaches.

## 1 Introduction

Time series classifiers are more and more often fundamental in a variety of important applications ranging from the identification of stock market anomalies to the automated detection of heart diseases (Bagnall et al., 2017). These tasks are automatized in Artificial Intelligence (AI) systems equipped with machine learning models and are adopted to support humans in sensitive decision-making processes. In the literature, there are various types of time series classifiers. In particular, the advent of deep architectures such as Convolutional (LeCun and Bengio, 1995) and Residual (He et al., 2015) Neural Networks has brought significant advantages in terms of accuracy and resistance to noise. Indeed, the best time series classifiers are proved to be Deep Neural Networks (DNNs) or ensemble-based classifiers (Ismail Fawaz et al., 2019). The drawback of these models lies in their complexity that makes them “black boxes” (Freitas, 2014) and causes the noninterpretability of the internal decision process for humans (Doshi-Velez and Kim, 2017). However, when important decisions, such as in clinical diagnosis, have to be taken, the *interpretability aspect* of AI performing Time Series Classification (TSC) becomes the crucial building block of a trustworthy interaction between the machine and the human expert. Therefore, only meaningful and interpretable TSC models can augment the cognitive ability of human experts, such as medical doctors, to make informed and accurate decisions (Pedreschi et al., 2019).

Recent research is focusing on developing explanation methods to pair with obscure time series classifiers adopted in AI systems (Schlegel et al., 2019; Guidotti et al., 2020). However, a TSC approach that is *intrinsically interpretable* and is simultaneously efficient and effective will be even more preferable because it is the whole model that results in being transparent by design (Guidotti et al., 2018) and does not require a subsequent explanation module that can be subject to errors or additional bias (Rudin, 2019). The idea is to aim for a classification model that would resemble the human way of thinking such that, as humans, we can entirely accept and trust the decision process automatically derived from machine learning algorithms. Indeed, for time series, there exists a line of research exploring interpretable time series classifiers based on *shapelets* (Ye and Keogh, 2009). Shapelet decision trees (Ye and Keogh, 2009) and shapelet transforms (Lines et al., 2012) extract the shapelets from the time series of the training set by selecting the subsequences with high discriminatory power and exploit them for the classification process. In Grabocka et al. (2014). an approach is proposed that extracts shapelet trees by solving an optimization problem. The weakness of these methods is that they are generally not very accurate or not sufficiently efficient because they require a large time to extract the shapelets and to train the classification model.

In this paper, we design MAPIC, a Matrix Profile- (MP-) based interpretable time series classifier. MAPIC is an interpretable model for TSC able to guarantee a high level of accuracy and efficiency while maintaining the classification and the classification model interpretable. To this aim, in the design of MAPIC, we follow the line of research based on shapelets. However, we replace the inefficient approaches adopted in the state of the art for the search of the most discriminative subsequences with the patterns that are possible to extract from a model named *Matrix Profile (MP)* (Yeh et al., 2016; Dau and Keogh, 2017; Mueen and Keogh, 2017). In short, the Matrix Profile represents the distances between all subsequences and their nearest neighbors. A MP is possible to efficiently extract some patterns characterizing a time series such as *motifs* and *discords*. Motifs are subsequences of a time series which are very similar to each other, while discords are subsequences of a time series which are very different from any other subsequence. As a classification model, MAPIC adopts a Decision Tree Classifier (dtc) (Quinlan, 2014) due to its intrinsic interpretability. Indeed, it is widely recognized that rule-based classifiers expressing the reasons for decisions in a logic form are among the most interpretable models (Doshi-Velez and Kim, 2017). However, we modified the learning of the decision tree in order to refine and reduce the search space for the best shapelet by (i) searching for the best shapelet for the split only among the motifs and discords of the time series present in the node being analyzed and (ii) only among *k* representative motifs and discords of these time series. We present experimentation on seventeen datasets of univariate time series with different dimensions, and we compare MAPIC against state-of-the-art interpretable classifiers based on shapelets and decision trees. We empirically demonstrate that MAPIC overtakes existing approaches having similar interpretability in terms of both accuracy and running time.

The rest of the paper is organized as follows. Section 2 discusses related works. Section 3 formalizes the problem faced and introduces basic concepts for the proposed interpretable classification model which is described in Section 4. Section 5 presents the experiments. Finally, Section 6 summarizes our contribution, its limitations, and future research directions.

## 2 Related Work

Given the need to accurately classify time series, researchers have proposed hundreds of methods to solve the TSC task (Bagnall et al., 2017). One of the most popular TSC approaches is the k-Nearest Neighbor (kNN) coupled with a distance function (Ismail Fawaz et al., 2019). The Dynamic Time Warping (DTW) distance has been shown to be a very strong baseline. Instance-based classifiers such as kNN can be considered interpretable classifiers. Indeed, one can adopt as an explanation the neighbors considered for distinguishing the class as for the prototypes and counterfactuals (Dhurandhar et al., 2018; Guidotti et al., 2019). The issues that can arise are the following. First, the inspection of the time series in the neighborhood could not sufficiently justify the classification and could be nontrivial when the neighborhood is large. Second, in case of large datasets, the kNN can be impractical to use in real applications, especially with the DTW. Third, an approach based on distances can have a partial vision of the TSC problem and have upper limits in the accuracy that can be reached.

Thus, recent contributions have focused on developing methods that outperform the kNN-DTW. In Lines and Bagnall (2015), COTE is shown, an ensemble of kNN classifiers with different distance functions that outperforms all the ensemble’s individual components. These approaches use either a Random Forest (Baydogan et al., 2013) or an ensemble of different types of classifiers on one or several feature spaces (Bagnall et al., 2015). These approaches significantly outperform the kNN-DTW (Bagnall et al., 2017) and share one common property, which is the data transformation where time series is transformed, for example, using shapelets (Bostrom and Bagnall, 2015) or SAX (Lin et al., 2003). In Lines et al. (2018). COTE is extended with a Hierarchical Vote system (HIVE-COTE) by leveraging a new hierarchical structure with probabilistic voting. HIVE-COTE is currently considered the nondeep learning state-of-the-art method for TSC (Bagnall et al., 2017). The increase in accuracy of these methods has simultaneously brought a further increase of the computational complexity (Ismail Fawaz et al., 2019) and a lack of interpretability due to the ensemble and the voting schemes (Guidotti et al., 2018).

The advent of deep architectures (LeCun and Bengio, 1995; He et al., 2015) has brought significant advantages in terms of accuracy and resistance to noise also for the TSC problem (Wang et al., 2017). In Ismail Fawaz et al. (2019), it is shown how DNNs are able to significantly outperform the kNN-DTW but are also able to achieve results that are not significantly different from COTE and HIVE-COTE. Although DNNs are more efficient at test time with respect to ensembles or to the kNN-DTW, the classification process of DNNs is not directly interpretable.

Another line of research on TSC explores interpretable methods based on *shapelets* (Ye and Keogh, 2009). Shapelet decision trees (Ye and Keogh, 2009) and shapelet transforms (Lines et al., 2012) extract the shapelets from the time series of the training set by selecting the subsequences with high discriminatory power and exploit them for the classification. In Grabocka et al. (2014). shapelets are learned, such that time series represented in their shapelet-transform space, i.e., their distances to each of the shapelets, are linearly separable. After that, the time series are represented as distances to shapelets, and any interpretable classifier such as decision trees or logistic regressors (Guidotti et al., 2018) can be used for the classification, also guaranteeing an explanation for the decision. These approaches are generally inefficient in finding the best shapelets and sometimes lack also in terms of accuracy. Reasons for possible inefficiencies of shapelets are discussed in detail in the following sections.

We advance the state of the art by proposing an interpretable method for TSC based on shapelets that reveal the decision process that is accurate and efficient.

## 3 Setting the Stage

Before presenting MAPIC, we go through some formal definitions for TSC and recall basic notions and key definitions necessary to comprehend the proposed method.

### 3.1 Problem Definition

Definition 3.1 (Time series) *A univariate time series* *x* = ⟨*x*_1_, *x*_2_, *…* , *x*_*m*_⟩ *is an ordered set of real values. The length of* *x* *is equal to the number of real values* |*x*| = *m* *.*

Definition 3.2 (TSC) *A dataset* *D* = {(*x* ^(1)^, *y* ^(1)^), *…*, (*x* ^(*n*)^, *y* ^(*n*)^)} *is a collection of pairs* (*x* ^(*i*)^, *y* ^(*i*)^) *where* *x* ^(*i*)^ *is a univariate time series with* *y* ^(*i*)^ *as its corresponding class label. For a dataset containing* *c* *classes,* *y* ^(*i*)^ *can take* *c* *different values. TSC consists of training a classifier* *f* *on a dataset* *D* *in order to map from the space of possible inputs to the space of possible classes.*

Our goal is to define a function *f* that is intrinsically transparent (Guidotti et al., 2018); i.e., it is humanly possible to understand the reasons for the decision process *f*(*x*) = *y*. Obviously, the interpretation is also highly dependent on the application and on the background of the user. Perhaps the average user can get some intuition related to the classification, while expert users can get the full picture. For instance, we can think of patients and doctors with respect to ECG analysis. In line with the literature (Ismail Fawaz et al., 2019), we assume that the time series in a dataset *D* have timestamps at the same sampling rate and are properly normalized such that geometric distances can be meaningfully calculated among them.

### 3.2 Shapelets

Shapelets are discriminative subsequences of time series that best predict the target class value (Ye and Keogh, 2009; Grabocka et al., 2014).

Definition 3.3 (Time series subsequence) *A time series subsequence (subsequence in short), of length* *l* *is an ordered subpart of a time series. A subsequence starting at time* *j* *inside the time series* *x* *is defined as* *x*_*j*,*l*_ = ⟨*x*_*j*_, *…* , *x*_*j*+*l*−1_⟩*. Thus, given a time series* *x* *of length* *m* *, there are* *q* = *m* − *l* + 1 *subsequences in* *x* *provided the starting index of a sliding window of length* *l* *is incremented by one.*

Definition 3.4 (Shapelets) *Given a TSC dataset* *D* *, a shapelet* *s* *is a subsequence of length* *l* *, with* *l* < *m* *where* *m* *is the length of the time series in* *D* *that maximizes the Information Gain when splitting* *D* *according to the distance between* *s* *and the time series in* *D* *with its corresponding optimal split point.*

In detail, the Information Gain is calculated as the difference between the entropy of the classes of the time series in *D* keeping *D* together or splitting it into two (or more) partitions. The optimal split point is the best distance threshold separating two partitions of time series to maximize the Information Gain. Further details can be found in (Ye and Keogh, 2009). In brief, a *shapelet* *s* is a subsequence of length *l* such that given a time series *x*, the distance of *s* from *x* can be used to infer how to discriminate the target class of a dataset *D*.

Definition 3.5 (Shapelets distance) *The distance between a times series* *x* *and a shapelet* *s* *is defined as the minimum distance* *dist* (*x*, *s*) *among the distances between* *s* *and each subsequence in* *x* *. In other words, it is the distance of a shapelet to the most similar subsequence* *:*

$$dist\left(x,s\right)=\min_{j=1,\ldots ,q}\frac{1}{l}\overset{l}{\underset{i=1}{\sum }}{\left(x_{j+i-1}-s_{i}\right)}^{2}$$

*where* *q* = *m* − *l* + 1*.*

Definition 3.6 (Shapelet transformation) *Given a set of* *k* *shapelets* *S* *and a set of* *n* *time series* *X* *, the shapelet transformation of* $X\in R^{n\times m}$ *into* $\hat{X}\in R^{n\times k}$ *is defined as the application of the distance* *dist* (*x*, *s*) *between all the time series* *X* *with all the shapelets* *S* *.*

In other words, every feature in $\hat{X}$ represents the minimum distance of a time series from a shapelet, which in turn is a subsequence with high discriminatory power. Therefore, a shapelet highlights the subparts of a time series that characterizes a specific class. In Hills et al. (2014). it is shown how general-purpose classifiers achieve high prediction accuracy over the shapelet transformation $\hat{X}$ .

### 3.3 Matrix Profile

A Matrix Profile is a data mining model that is possible to extract on a single time series (Yeh et al., 2016). In short, it represents the distances between all subsequences existing in a time series given certain sliding window size and their nearest neighbors in the same series.

Definition 3.7 (All-subsequences set) *An all-subsequences set* *A* *of a time series* *x* *is an ordered set of all possible subsequences of* *x* *obtained by sliding a window of length* *l* *across* *x* *:* *A* = {*x*_1,*l*_, *…* , *x*_*q*,*l*_}*, where* *l* *is a user-defined subsequence length and* *q* = *m* − *l* + 1*.*

Definition 3.8 (Distance profile) *The distance profile* *P*_*z*_ *of a time series* *x* *for a query subsequence* *z* *is a vector of the distances from* *z* *and each subsequence in* *A* *of* *x* *:*

$$P_{z}=\left\langle p_{1},\ldots ,p_{q}\right\rangle s.t.p_{j}=\frac{1}{l}\overset{l}{\underset{i=1}{\sum }}{\left(x_{j+i-1}-z_{i}\right)}^{2}\;\forall j=1,\ldots ,q$$

*where* *q* = *m* − *l* + 1*.*

Definition 3.9 (Matrix Profile) *The matrix profile* *M* *of a time series* *x* *is a vector where the* *j* *-th value* *m*_*j*_ *represents the distance between the subsequence* *x*_*j*,*l*_ *and the closest subsequence to* *x*_*j*,*l*_ *in* *x* *different from* *x*_*j*,*l*_ *, i.e., the minimum distance in* $P_{x_{j,l}}$ *without considering* *x*_*i*,*j*_ *aligned with itself at position* *j* *:*

$$M=\left\langle m_{1},\ldots ,m_{q}\right\rangle s.t.m_{j}=\min\left\{P_{x_{j^{\prime },l}}\;|\;\forall j^{\prime }=1,\ldots ,q\wedge j^{\prime }\neq j\right\}\;\forall j=1,\ldots ,q$$

*where* *q* = *m* − *l* + 1*.*

We highlight that we assume that all the subsequences have length *l*. However, theoretically, multiple MP with different lengths can be extracted and considered. We leave it as future work.

A MP can be considered as a *meta* time series with various interesting properties. The minimum values in a MP correspond to the locations of the best time series *motif* pair (Mueen et al., 2009). Motifs are subsequences of a time series that are very similar to each other. On the other hand, the maximum values in a MP correspond to *discords* (Chandola et al., 2009), i.e., subsequences of a time series that are very different from any other subsequence.

## 4 Matrix Profile-Based Interpretable Classifier

MAPIC is a Matrix Profile-based interpretable time series classifier. The main intuition behind MAPIC is the following. First, to find the best shapelets, MAPIC exploits the Matrix Profiles extracted from the time series of the training set instead of using a brute force approach (Ye and Keogh, 2009) or an optimized search (Grabocka et al., 2014). As candidate shapelets, MAPIC adopts the motifs and discords that are possible to retrieve from the Matrix Profiles of each time series. Second, differently from traditional approaches that learn machine learning models for TSC directly on all the shapelet transformation (Grabocka et al., 2014), MAPIC builds a decision tree by refining at each split the set of candidate shapelets that better represent the times series in the current split. Algorithms 1 **,** 2 illustrate the pseudocode of MAPIC. Details are in the following.

**Algorithm 1:** MAPIC (*D*, *l*, *h*, *k*, *max*_*depth*)

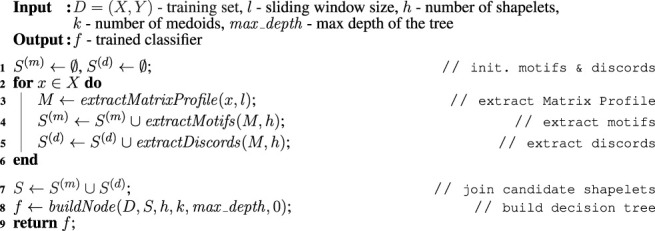

**Algorithm 2:** buildNode (*D*, *S*, *h*, *k*, *max*_*depth*, *cur*_*depth*)

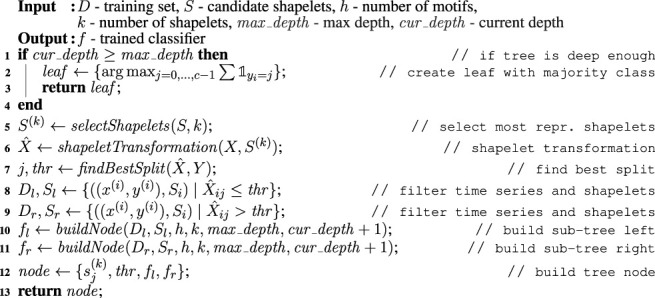

### 4.1 Matrix Profile Extraction

MAPIC takes as input the time series training set *D* = {*X*, *Y*}, the sliding window size *l*, the number of shapelets *h*, the number of medoids *k*, and the maximum depth of the tree *max*_*depth* and returns as output a time series classifier *f*: *X* ^*m*^ → [0, *c* − 1] in the form of dtc. MAPIC starts by initializing two empty sets of candidate shapelets (line 1, Algorithm 1): *S* ^(*m*)^ collects the motifs, while *S* ^(*d*)^ collects the discords. Then, for each time series in the training set *X*, MAPIC extracts the Matrix Profile *M* using a sliding window size *l* and from *M* retrieves *h* motifs and *h* discords (lines 2–6, Algorithm 1). After that, it joins *S* ^(*m*)^ and *S* ^(*d*)^ in the set of all the possible candidate shapelets *S* and starts the construction of the decision tree based on Matrix Profile and shapelets (lines 7–8, Algorithm 1). Finally, it returns *f*.

### 4.2 Tree Construction

As a classification model, MAPIC adopts dtc (Quinlan, 2014). It is worth mentioning the existence of other approaches based on inductive logic programming such as those presented in (Law et al., 2021) and (D’Asaro et al., 2020) which, in some context, can be more accurate and transparent than decision trees. However, among machine learning approaches, we decided to adopt a decision tree due to its intrinsic interpretability (Breiman et al., 1984; Craven and Shavlik, 1995) that allows us to reason both “factually,” deriving the reasons why a certain decision is taken, and “counterfactually,” observing what should have been dissimilar to reach a different outcome (Guidotti et al., 2019). Besides, it is widely recognized that machine learning rule-based classifiers are among the most interpretable models (Doshi-Velez and Kim, 2017).

The *buildNode* function, illustrated in Algorithm 2, is called recursively by MAPIC to build the dtc. The recursion ends when the depth of the tree is higher than *max*_*depth* (line 1, Algorithm 2) or other classic stopping conditions, not reported here for the sake of simplicity, are met. In this case, a leaf is returned indicating the majority class among the instances *y* ^(*i*)^ ∈ *Y* in the current split (lines 2–3, Algorithm 2). The $1_{y^{\left(i\right)}=j}$ operator returns 1 when the *i*-th time series is of class *j*. The arg max reveals the majority class.

In line five of Algorithm 2, MAPIC selects with the *selectShapelets* function *k* shapelets to represent the dataset *D* among those in the current set of candidate shapelets *S* (if |*S*| > *k*). This selection allows considerably reducing the search space for the best shapelets from |*S*| to *k* with *k* ≪|*S*| and avoiding useless calculations. Indeed, a preliminary empirical study has shown that many of the candidate shapelets obtained from motifs and discords of the Matrix Profile are quite similar to each other. Thus, it is not necessary to consider all of them. This similarity is even stronger if we only consider the motifs and discords of the shapelets in the current split^1^. We implement the *selectShapelets* function through the K-medoids algorithm (Schubert and Rousseeuw, 2019). K-medoids is a variant of K-means (Lloyd, 1982) where medoids are used instead of centroids. While a centroid is obtained as the average of the features’ values, a medoid is an instance in a cluster that minimizes the distance with all the other instances in the same cluster. We preferred K-medoids to K-means because the medoids selected as most representative shapelets candidates are indeed real subsequences, i.e., motifs or discords in *S*, and not the result of an aggregation that could also be not representative. We indicate with *S* ^(*k*)^ the set of *k* shapelets selected by K-medoids. After that, MAPIC calculates the shapelets transformation $\hat{X}$ on the most representative shapelets *S* ^(*k*)^ and then finds the best split (lines 6-7 of Algorithm 2) using the *findBestSplit* function. In line with classic algorithms for the induction of decision trees such as ID3 (Quinlan, 1986) and C4.5 (Quinlan, 2014), the *findBestSplit* function finds the best split among the *k* continuous attributes adopting the entropy (Shannon, 1948) as impurity measure. The pair *j*, *thr* models, respectively, the index *j* of the shapelet in the shapelets transformation $\hat{X}$ and the threshold value *thr* that better separates the labels *Y* between two different classes. In other words, the lowest level of impurity for the dataset *D* with respect to the shapelets *S* ^(*k*)^ is reached when separating the times series *x*_*i*_ ∈ *X* having a distance from the *j*-the shapelet in *S* ^(*k*)^ (namely, $s_{j}^{\left(k\right)}$ ) lower than or equal to *thr*, i.e., ${\hat{X}}_{ij}=dist\left(x_{i},s_{j}^{\left(k\right)}\right)\leq thr$ from the times series *x*_*i*_ ∈ *X* having a distance higher than *thr*, i.e., ${\hat{X}}_{ij}=dist\left(x_{i},s_{j}^{\left(k\right)}\right)>thr$ . Therefore, MAPIC splits the dataset *D* and the candidate shapelets *S* into the disjoint sets *D*_*l*_, *S*_*l*_ and *D*_*r*_, *S*_*r*_ respecting the aforementioned conditions for building the left and right subtrees, respectively (lines 8–9 Algorithm 2). The recursion takes place in recursively building the left and right subtrees *f*_*l*_, *f*_*r*_ in the left and right partitioning *D*_*l*_, *S*_*l*_, and *D*_*r*_, *S*_*r*_ (lines 10–11 Alg. 2). Finally, in line 12 Alg. 2, an internal node of the tree is built and returned. It includes the best shapelet $s_{j}^{\left(k\right)}$ for the current split, the distance threshold *thr*, and the left and right subtrees *f*_*l*_, *f*_*r*_.

As already mentioned, we stress that decision trees traditionally trained on shapelets transformation consider the same set of shapelets at every iteration. Thus, the training procedure is conditioned from the initial search of the shapelets. On the other hand, MAPIC considers as candidate shapelets for every split only the motifs and discords of the time series belonging to the current split. This allows for a locally refined search of the best shapelet that, as shown in the experimental section, markedly improves the performance.

### 4.3 Prediction Procedure

At prediction time, the MAPIC classification function *f* takes as input the time series $x\in R^{m}$ and returns the label *y* = *f*(*10*) according to the following procedure. It starts from the root of the tree and calculates the shapelet transformation w.r.t. the shapelet *s* in the root node, i.e., *dist* (*x*, *s*_*root*_). If *dist* (*x*, *s*_*root*_) ≤ *thr*_*root*_, then the classification is repeated on the left subtree classification function *f*_*l*_, otherwise, on the right subtree classification function *f*_*r*_. The prediction procedure terminates when a leaf is reached and the label *y* in the class is returned as a final outcome.

### 4.4 Computational Complexity

We conclude this section by analyzing the complexity of MAPIC and how the usage of the Matrix Profile and of K-medoids is helpful in reducing the overall complexity. In order to better analyze the computational complexity of the proposed method and to compare it with those of the approaches in the literature (Ye and Keogh, 2009; Grabocka et al., 2014), we decompose our reasoning into different phases: *(i)* shapelets extraction, *(ii)* tree construction, *(iii)* shapelets transformation, and *(iv)* TSC. Depending on the method, considered different phases take place at training or at prediction time. We recall that, in our notation, *n* indicates the number of time series, *m* is the time series length, *l* is the sliding window size, *k* is the number of shapelets extracted, and *h* is the number of motifs/discords. We indicate with *depth* the depth of the decision tree and with *iter* the number of iterations of a certain procedure.

Independently from the algorithm adopted, the shapelet extraction is the step with the highest computational complexity among the various phases. Since the size of a candidate set is *O* (*n* ⋅ *m* ^2^), the brute force (bf) method which exhaustively tries candidates from series segments (Ye and Keogh, 2009; Lines et al., 2012) takes *O* (*n* ⋅ *m*) to check the utility of one candidate results in a complexity of *O* (*n* ^2^ ⋅ *m* ^3^). On the other hand, the approach proposed in (Grabocka et al., 2014) (opt) requires *O* (*n* ⋅ *m* ^2^ ⋅*iter*). With respect to MAPIC, the brute force procedure for the extraction of a Matrix Profile requires *O* (*m* ^2^ ⋅ *l*). However, according to (Yeh et al., 2016), when possible, we adopt the STOMP algorithm which requires *O* (*m* ^2^ ⋅  log(*m*)) that empirically results to be *O* (*m* ^2^). The cost of extracting motifs and discord is linear with the number required *O*(*h*) and negligible with respect to the MP extraction. Thus, repeating the MP extraction for every time series results in computational complexity of *O* (*n* ⋅ *m* ^2^ ⋅  log(*m*)) that is better than opt, the shapelet-based classifier based on optimization to extract shapelets, when log(*m*) < *iter*. We can claim that it is always true considering that, in (Grabocka et al., 2014), the procedure typically converges after no more than 1,000 iterations. The tree construction phase requires *O* (*n* ⋅ *m* ⋅*depth*) for a decision tree learning on the whole time series and *O* (*n* ⋅ *k* ⋅*depth*) for a decision tree learning on a dataset represented by the distances with *k* shapelets. In addition, both bf and opt require a shapelet transformation with a cost of *O* (*n* ⋅ *m* ⋅ *k*) before the tree construction, while MAPIC repeats the shapelet transformation several times during the tree construction but pays *O* (*n* ⋅ *m* ⋅ *k*) only for the first split. Therefore, these costs are comparable^2^. MAPIC additionally pays the complexity of K-medoids that is *O* (*n* ⋅ *m* ⋅ *k* ⋅*iter*). However, it gains a benefit at prediction time. Indeed, when using bf or opt to classify an unseen time series, a shapelet transformation must be run. This transformation cost *O* (*m* ⋅ *k*). On the other hand, for MAPIC, it only costs *O* (*m* ⋅*nodes*) where *nodes* = 2^*depth*^ − 1 indicates the number of internal nodes of a balanced tree. Indeed, due to its procedure that is driven by local choices for every node, in the end, MAPIC only accounts for the shapelets present in the tree discarding all the others considered during the training. Since in (Grabocka et al., 2014) *k* is selected as a percentage w.r.t. the time series length, on average, MAPIC is the lowest complexity at prediction time when *m* > 50 which is often the case.

### 4.5 Limitations

The proposed MAPIC is largely based on the concept of shapelets. Despite their not negligible interpretability, approaches based on shapelets can suffer from inefficiencies in terms of accuracy. These inefficiencies can be due to several different reasons. As their name suggests, shapelets are typically suitable when time series can be separated among classes looking at the shape of some subparts. Therefore, they characterize through “local” aspects of the time series of the dataset under analysis how to discriminate among classes. However, if the characterizing aspects for a class are more “global,” then shapelets can fail, leading to poor performance. Similarly, from an interpretability perspective, an expert would more likely agree on classifying a time series with respect to a global criterion like the average value or variance rather than the shape of its subsequences. In light of this reasoning, MAPIC is naturally more suitable for applications where the aim is to recognize the class of a time series based on local shapes. However, MAPIC can also be extended by also considering global features like mean, variance, and trend in parallel with the shapelets distances to build the decision tree. We leave this extension as future work. Also, being based on local features, shapelets can be theoretically affected by scale transformations. However, in our proposal, shapelets are obtained from motifs and discords of the MP that automatically preprocess the data (Yeh et al., 2016). Therefore, thanks to the MP, MAPIC is executed on the input provided without requiring any special preprocessing.

## 5 Experiments

In this section, we show the interpretability, accuracy, and efficiency of MAPIC on various datasets and against several interpretable state-of-the-art time series classifiers^3^. The rest of this section is organized as follows. First, we illustrate the experimental setting. Then, we provide a qualitative evaluation of the interpretable classification offered by MAPIC through a visual inspection of a shapelet-based decision tree. After that, we compare MAPIC against state-of-the-art interpretable time series classifiers. Finally, we discuss the effect of the parameters on the performance of MAPIC.

### 5.1 Experimental Setting

We experimented with MAPIC on 17 datasets usually adopted for benchmarking in univariate TSC having various numbers of instances, lengths, and number of classes. Table 1 reports the details. In order to ensure a fair comparison with the baselines, we used the default train and test data splits (Lines et al., 2012; Hills et al., 2014). The datasets are available through the UCR website^4^.

**TABLE 1:** Datasets details: *n* number of time series in the dataset, *m* time series length, and *c* number of classes.

	*n*	*m*	*c*	Training (%)	Test (%)
ArrowHead	211	251	3	17	83
BirdChicken	40	512	2	50	50
Coffee	56	286	2	50	50
Earthquakes	461	512	2	70	30
ECG200	200	96	2	50	50
ECG5000	5,000	140	5	10	90
FaceFour	112	350	4	21	79
GunPoint	200	150	2	25	75
PowerDemand	1,096	24	2	6	94
Phalanges	2,658	80	2	68	32
Strawberry	983	235	2	62	38
Trace	200	275	4	50	50
TwoLeadECG	1,162	82	2	2	98
Wafer	7,164	152	2	14	86
Wine	111	234	2	51	49
WormsTwoClass	258	900	2	70	30
Yoga	3,300	426	2	9	91

We compared MAPIC against the following interpretable time series classifiers. Since we argue that decision trees are among the most interpretable models, we focus on classifiers adopting this structure and we differentiate the procedure used to extract shapelets. In particular, we adopt the brute force approach (bf) presented in (Ye and Keogh, 2009) and the one based on optimization (opt) presented in (Grabocka et al., 2014). Besides, we equip a dtc with the motifs and discords extracted with the first step of MAPIC. We name this approach Matrix Profile Decision Tree (mpdt) as it differs from MAPIC from the internal (local) selection of candidate shapelets. Finally, we compare against two traditionally interpretable models not using shapelets: a kNN and a dtc using as features the timestamps of the time series^5^. In the following, we use the term *completely interpretable* classifiers to refer to the shapelet-based approaches MAPIC, bf, opt, and mpdt, while *noncompletely interpretable classifiers* to indicate dtc and kNN. We adopt this description because, as shown in the qualitative evaluation section, through completely interpretable classifiers, it is possible to fully understand which are the subparts of the time series causing the classification, while with noncompletely interpretable classifiers this is not immediately possible. We stress the fact that we do not compare MAPIC against deep learning-based solutions, solutions based on time series approximation (such as SAX), or alternative representations (like the Fourier one) as this is beyond the purpose of this paper. Our aim is to compare against shapelet-based classifiers. Hence, we do not compare also against time series classifiers based on structural features such as mean, min, and max.

We highlight that, in TSC, kNN and dtc are not interpretable as the other methods. Indeed, kNN requires inspecting the time series in the neighborhood to find similarities with the one classified and this can be not trivial if the neighborhood is large or if the time series is particularly long. On the other hand, dtc identifies as features for the tree individual time stamps along the time series that do not reveal as clearly as shapelets the classification reasons.

After empirical experimentation, we adopted the following parameter settings that have the best results for the various methods. For every decision tree, we use *entropy* as a gain criterion. In our opinion, not every decision tree is interpretable. Indeed, we believe that a decision tree is not interpretable if it is too complex, i.e., too deep. Hence, for the sake of interpretability, we do not grow each tree with a depth higher than 3 and we guarantee that each leaf has at least 20 records in a node. For the shapelets, we adopt a sliding window *l* = 20. For MAPIC and mpdt, we use *h* = 3 discords per time series and *k* = 20 shapelets. For bf and opt, the default parameters of the libraries are used. For kNN, we use *k* = 3.

As an evaluation metric, we observed the classification *accuracy* and the *running time* expressed in seconds.

### 5.2 Qualitative Evaluation

**FIGURE 1:**
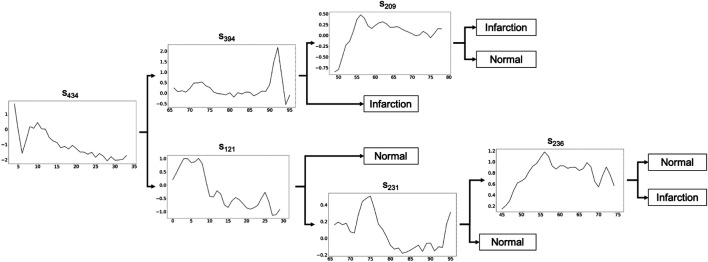
Shapelet-based tree obtained by MAPIC on ECG200. If the time series classified has a distance to the shapelet in the node smaller than the threshold (not reported), the classification moves on the upper part, otherwise on the lower part.

In this section, we show the application of MAPIC^6^ for a possible real case usage on the ECG200 dataset (Olszewski, 2001) with a focus on the global and local interpretability of the model. The goal for ECG200 is to distinguish time series representing heart rate between *normal heartbeat* and *myocardial infarction*. The tree returned by MAPIC is illustrated in Figure 1. If the time series classified has a distance to the shapelet in the node smaller than the threshold (not reported on the tree), then the classification moves on the upper part of the tree, otherwise on the lower part. In particular, we notice how in the upper part it is possible to distinguish the presence of infarction if the first discriminative shapelet *s*_434_ is present in a time series and the second one (*s*_394_) is not. This reveals that a jump from a high value to a lower one and vice versa is a clear indication of a possible infarction. On the other hand, if another jump from a low value to a high one and vice versa is present, then the risk of infraction is lower. A similar reading can be done on the lower part of the tree. This transparent and global vision of the logic of the AI system adopting a MAPIC tree can be useful to practitioners. Indeed, it can reveal how the AI system is reasoning and agree with it or not in an easier way. Also, developers can unveil misclassification reasons and vulnerabilities and act to align the AI reasoning with human believes.

**FIGURE 2:**
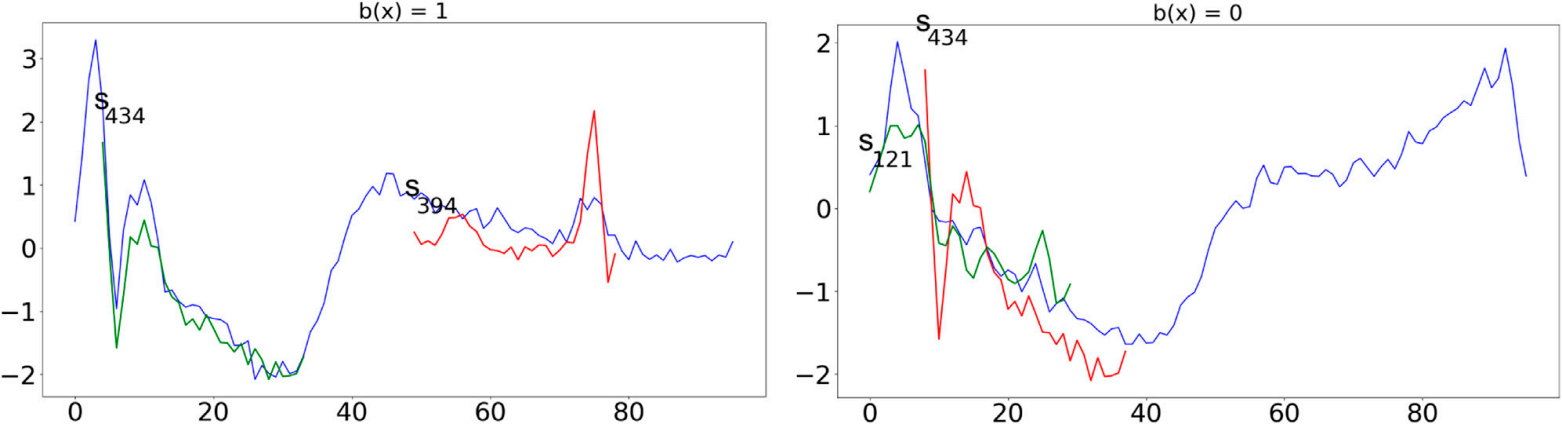
Application of the tree in Figure 1 to classify two time series with class infarction **(left)** and normal **(right)**. Shapelets are always shown at their best alignment with the time series and are colored in green if they are “contained” in the time series (i.e., the distance between the series and the shapelet is slower than the threshold), in red otherwise.

In Figure 2, we show an application of the tree to classify two instances with class infarction (left) and normal (right). The shapelets are always shown at their best alignment with the time series and are colored in *green* if they are “contained” in the time series (i.e., the distance between the series and the shapelet is slower than the threshold), in *red* otherwise. The time series on the left respect exactly the infarction pattern described above by containing *s*_434_ and not containing *s*_394_. On the other hand, the time series on the right, which is similar in shape to the one on the left, does not contain *s*_434_ and contains *s*_121_ indicating a “normal” decrease of the value and therefore a normal heartbeat.

We believe that this visual inspection clearly illustrates the effectiveness of the transparent classifiers returned by MAPIC. However, since similar models can be obtained with state-of-the-art approaches, in the next section, we show how MAPIC overperforms them.

### 5.3 Quantitative Comparison

In the following, we discuss the results obtained by comparing MAPIC with the interpretable time series classifiers illustrated in the experimental setting section.

Table 2 reports the accuracy of MAPIC and of the other methods analyzed. We notice that, among the completely interpretable time series classifiers adopting shapelet-based decision trees, MAPIC has on average the highest performance. In particular, MAPIC has the highest accuracy on 11 out of 17 datasets, and it is the second-best performer for the other five datasets. Indeed, the average ranking of MAPIC is 2.38 also considering the nonshapelet-based classifiers. Among the competitors, bf is generally the second-best performer among shapelet-based classifiers, while kNN is the overall second-best performer with the highest average accuracy but with the aforementioned interpretability problems.

**TABLE 2:** Accuracy of MAPIC and competitors. Among shapelet-based classifiers, bold indicates the best performer and italic the second one (the higher the better).

Dataset	MAPIC	mpdt	opt	bf	dtc	knn
ArrowHead	**0.53**	0.39	0.39	0.39	0.39	0.64
BirdChicken	**0.80**	0.50	0.50	0.50	0.50	0.45
Coffee	**0.96**	0.54	0.54	0.54	0.54	0.89
Earthquakes	*0.74*	0.73	**0.75**	0.71	0.68	0.74
ECG5000	**0.91**	0.46	**0.91**	0.90	0.90	0.87
ECG200	**0.78**	0.64	0.68	**0.78**	0.76	0.85
FaceFour	**0.24**	0.16	0.16	0.16	0.16	0.68
GunPoint	**0.91**	0.73	0.71	*0.75*	0.63	0.85
Phalanges	0.65	0.62	*0.67*	**0.68**	0.69	0.66
PowerDemand	*0.92*	0.53	0.61	**0.94**	0.97	0.93
Strawberry	*0.87*	0.64	0.85	**0.90**	0.88	0.94
Trace	**0.98**	0.66	0.89	**0.98**	0.72	0.74
TwoLeadECG	**0.77**	0.50	0.50	0.50	0.50	0.58
Wafer	**0.95**	0.89	0.92	**0.95**	0.97	0.99
Wine	*0.61*	0.50	**0.74**	0.57	0.74	0.72
WormsTwoClass	*0.68*	0.43	0.60	**0.71**	0.52	0.61
Yoga	**0.66**	0.52	0.61	*0.62*	0.60	0.79
Avg	**0.76**	0.55	0.64	*0.68*	0.65	0.76
Rank	**2.38**	5.26	3.79	*3.26*	3.79	2.50

In Table 3, we report the training (left) and prediction (right) times, respectively. Concerning training time, it shows us that MAPIC is second in training time only to mpdt among the shapelet-based decision trees. The additional training time of MAPIC over mpdt for locally running the shapelets selection procedure, i.e., K-medoids in our implementation, is justified by the significant increase of the accuracy. On the other hand, opt and bf have a higher training time, which is generally one order of magnitude higher for opt and two orders of magnitude higher for bf which makes an exhaustive search of the shapelets. With respect to prediction time, we observe that opt has the lowest prediction time^7^, but MAPIC is nearly always the second-best performer among the shapelet-based classifiers. Finally, the noncompletely interpretable classifiers dtc and kNN have training and prediction times markedly lower than the shapelet-based ones. These higher times are due to the calculus of the distance between the time series and the shapelets.

**TABLE 3:** Training time (left) and prediction time (right) in seconds of MAPIC and competitors. Among shapelet-based classifiers, bold indicates the best performer and italic the second one (the lower the better).

Dataset	Training time						Prediction time					
	MAPIC	mpdt	opt	bf	dtc	knn	MAPIC	mpdt	opt	bf	dtc	knn
ArrowHead	*6.31*	**2.65**	43.09	35.69	<0.01	0.01	*0.56*	5.07	**0.06**	6.44	<0.01	0.03
BirdChicken	*3.10*	**2.37**	56.15	34.24	<0.01	0.01	**0.04**	0.60	**0.04**	25.86	<0.01	0.02
Coffee	*3.48*	**2.23**	32.66	22.93	<0.01	0.01	**0.04**	0.81	**0.04**	7.66	<0.01	0.01
Earthquakes	1539.07	**1335.27**	*416.79*	4654.63	0.06	0.05	6.52	25.98	**0.06**	*2.77*	0.01	0.08
ECG5000	*86.28*	**29.22**	123.38	982.01	0.04	0.02	42.63	140.22	**0.37**	*8.83*	0.04	0.93
ECG200	*16.11*	**4.70**	31.08	27.15	<0.01	<0.01	*0.58*	2.75	**0.05**	1.68	<0.01	0.01
FaceFour	49.01	*44.98*	45.85	**31.78**	<0.01	0.01	*0.64*	11.62	**0.05**	11.75	<0.01	0.02
GunPoint	*7.46*	**2.82**	28.91	19.90	<0.01	0.01	*0.68*	4.18	**0.05**	3.04	<0.01	0.02
Phalanges	647.55	*217.69*	**199.37**	3552.25	0.07	0.05	15.46	43.92	**0.09**	*1.71*	0.01	0.24
PowerDemand	6.89	*2.23*	26.67	**0.76**	<0.01	<0.01	2.74	24.24	**0.10**	*0.83*	<0.01	0.05
Strawberry	*122.82*	**43.59**	236.84	3821.50	0.06	0.05	*4.29*	11.70	**0.07**	6.57	0.01	0.12
Trace	*19.70*	**7.56**	67.65	192.35	0.01	0.01	*0.61*	2.92	**0.05**	7.26	<0.01	0.02
TwoLeadECG	*2.39*	**1.09**	22.81	3.01	<0.01	0.01	1.88	30.73	**0.10**	*1.80*	0.01	0.07
Wafer	576.50	*359.74*	**213.21**	4140.29	0.06	0.07	129.88	431.18	**0.49**	*12.91*	0.06	1.59
Wine	*9.85*	**3.88**	44.13	48.80	<0.01	0.01	0.40	1.52	**0.04**	*0.21*	<0.01	0.02
WormsTwoClass	*58.31*	**34.94**	671.60	4772.95	0.05	0.06	*0.90*	2.51	**0.07**	4.69	0.01	0.07
Yoga	*77.09*	**30.16**	294.04	2855.68	0.05	0.05	*19.76*	94.55	**0.49**	59.70	0.08	0.87
Avg	*109.11*	**124.00**	150.25	1482.11	0.02	0.03	*13.39*	49.08	**0.13**	9.63	0.01	0.24
Rank	*4.35*	**3.29**	5.05	5.29	1.35	1.64	*4.38*	5.64	**2.64**	4.94	1.0	2.38

**FIGURE 3:**
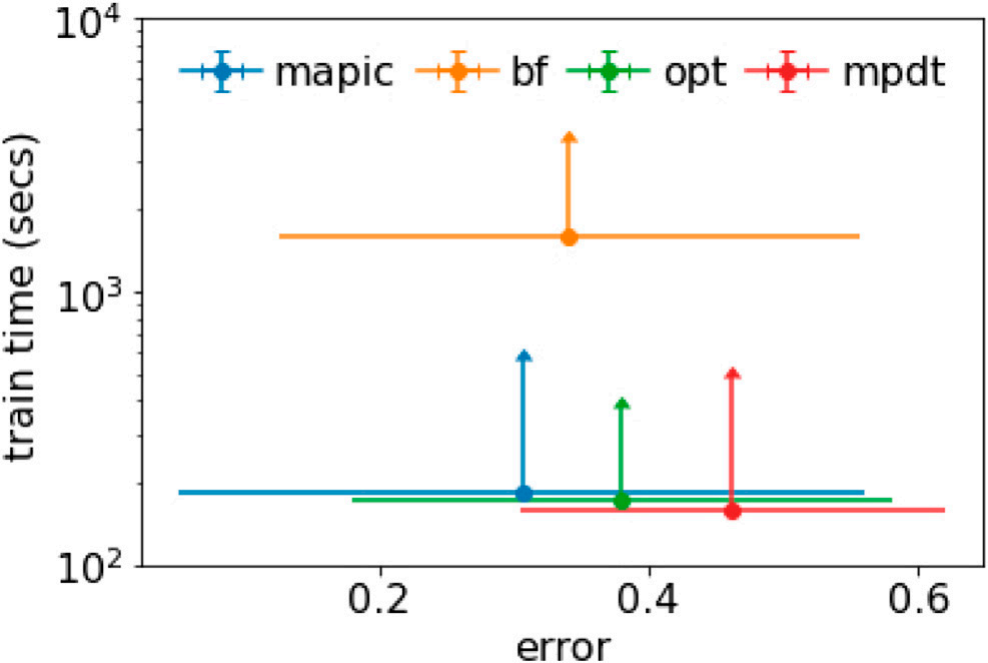
Scatter plot with error bars for average and std. dev. of error and training time (in log scale).

We summarize and combine the results in Figure 3 where we consider simultaneously the accuracy (expressed as error, i.e., 1-accuracy) and the training time. Figure 3 depicts a scatter plot reporting the average values together with error bars representing the standard deviation. We notice that MAPIC lies in the bottom-left part of the graphics indicating the lowest error and lowest training time. We remark that the training time is in a logarithmic scale.

**FIGURE 4:**
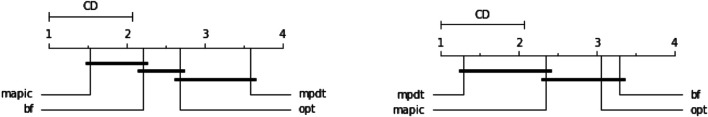
Critical difference diagrams using the post hoc Nemenyi test with *α* = 0.05 to display statistical differences in terms of accuracy **(left)** and training time **(right)**.

Moreover, we represent the comparison of the ranks of all explainers against each other in Figure 4 with the critical difference plots (Demšar, 2006). Critical difference diagrams show the results of a statistical comparison of the performance of the methods. In these diagrams, the methods, represented by vertical plus horizontal lines, are displayed from left to right in terms of the average rank obtained for the various evaluation measures and experiments^8^. Horizontal bold lines connect the methods producing statistically equivalent performance according to a post hoc Nemenyi test with *α* = 0.05. We observe that MAPIC is statistically the best performer for accuracy with respect to mpdt and opt, while it is the second-best performer with respect to training and prediction time. Even though there is not a markedly clear statistical significance between MAPIC and all the competitors, we notice that also all the other methods are always tied. Therefore, MAPIC results show its empirical superiority with respect to the other methods.

### 5.4 Sensitivity Analysis

**FIGURE 5:**
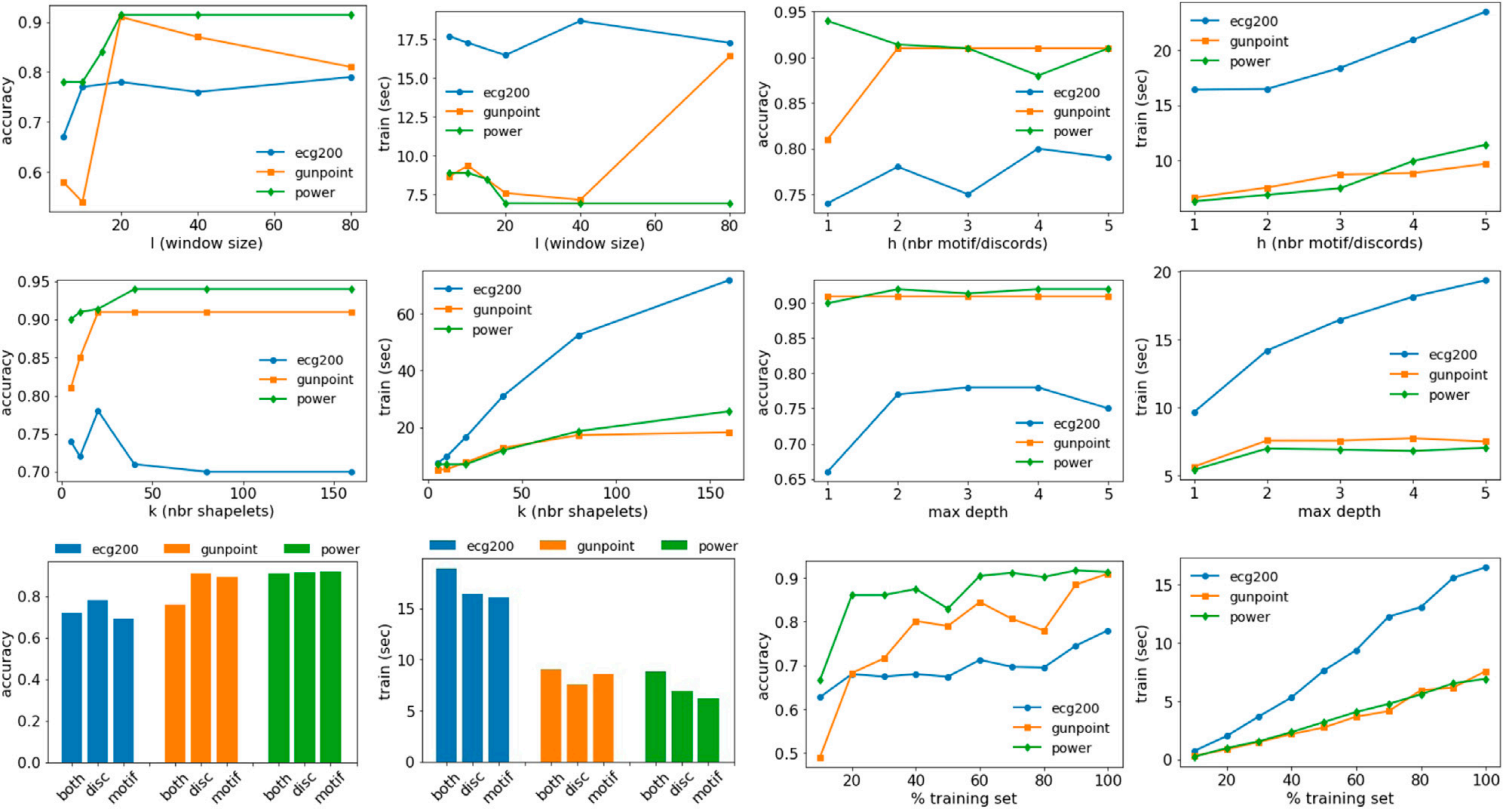
Effect of the parameters on the accuracy and training time of MAPIC. First row: window size (*l*) and number of motifs/discords (*h*). Second row: number of shapelets (*k*) and max depth of the decision tree. Third row: usage of motifs/discords or both and training set size.

In this section, we discuss the effect of the parameters on the accuracy and training time of MAPIC. In particular, we analyze the effect of the window size *l*, the number of motifs/discords *h*, the number of shapelets considered *k*, the maximum depth *max*_*depth* of the tree, the usage of motifs and/or discords, and the percentage of the training set. Figure 5 shows all the results for the datasets ECG200, GunPoint, and PowerDemand. The window size *l* impacts the accuracy but not necessarily the training time. It generally reaches a plateau for *l* ≥ 20. The number of motifs/discords *h* slightly increases the training time but has a small effect on the accuracy, and *h* > 1 motifs/discords are sufficient to guarantee the performance illustrated in the previous section. On the other hand, the number of shapelets *k* considered in each split and filtered using the selection procedure impacts both accuracy and training time. On average, for *k* ∼ 20, we observed the best results without a too high time overhead. The maximum depth of the tree does not particularly affect the accuracy, while higher values can increase the training time. With respect to motifs and/or discords, there is not a clear pattern for accuracy; therefore, we preferred to report results only using discords that, on average, reach higher performance. Using both motifs and discords increases the training time. The percentage of the training set used impacts more on the training time with an increase slightly less than linear. On the other hand, competitive levels of accuracy are also reached using small portions of the training data starting from 20%. We conclude by reporting some statistics related to the number of iterations of the K-medoids procedure that can highly impact the computational complexity. Among all the datasets analyzed, we have on average 4 ± 2.13 iterations with a minimum of 2 and a maximum of 8. Therefore, in practice, the usage of K-medoids is negligible in terms of complexity.

## 6 Conclusion

We have presented MAPIC, an interpretable model for TSC based on Matrix Profile, shapelets, and decision tree. The usage of the Matrix Profile allows MAPIC to reduce the cost for retrieving the best shapelets by adopting motifs and discords. A further speed-up is given by the usage of K-medoids to avoid analyzing too similar candidates. Finally, the shapelet-based decision tree provides intrinsic global interpretability that can be easily extended for specific classification cases analyzing the path from the root to the leaves. Wide experimentation on various datasets and against state-of-the-art interpretable time series classifiers reveals that MAPIC is statistically competitive with existing approaches or overcomes them.

The method has some limitations. The sliding window parameter constrains the shapelets to a predetermined size. According to the state of the art (Senin and Malinchik, 2013), this issue can be easily overcome by repeating the Matrix Profile extraction with sliding windows of different sizes. The shapelet-based rules do not consider multiple alignments of the same shapelet at different points of the time series. However, multiple occurrences could help in better explaining a predictive phenomenon. Several future research directions are possible. MAPIC only works for univariate and multiclass time series classifiers. A challenge is to extend MAPIC to make it also work for multivariate time series and multilabel classification. Also, technical and conceptual extensions are possible: first, extending MAPIC such that it can work on different types of sequential data like text or shopping transactions through the adoption of time series approximation; second, by enabling MAPIC to automatically account for model complexity while building the decision tree in order to avoid parameters related to the generalization of the model, and finally, a real improvement with respect to the current evaluation which would be an experiment involving humans testing to which extent the decision trees returned by MAPIC can be considered “transparent” and valuable. Our idea is to conduct extrinsic interpretability evaluation asking humans experts in a certain area, e.g., medical doctors, to assess specific tasks driven by MAPIC results. In such a way, we can simultaneously survey the usability of MAPIC through the opinions of the experts and also objectively and quantitatively evaluate the success rate in the tasks assigned.

## Data Availability

The original contributions presented in the study are included in the article/Supplementary Material; further inquiries can be directed to the corresponding author.

## Footnotes

1 The median average sum of distances between all the shapelets available in a node and those selected among various iterations for all the datasets analyzed in the experiment section is lower than 20.

2 Both costs should be multiplied by l to account for the Euclidean distance. We omit it as it does not impact the complexity.

3 Code available at: https://github.com/matteo-Donofrio-unipi/Mapic. MAPIC is realized using a revised version of the matrixprofile-ts library https://github.com/target/matrixprofile-ts for extracting the Matrix Profile, the libraries binarytree https://pypi.org/project/binarytree/ and scikit-learn https://scikit-learn.org/ for the implementation of the shapelet-based decision tree, and scipy https://docs.scipy.org/doc/scipy/reference/ for the calculus of the distances. Experiments were run on Windows 10, Intel i5-3570K 3.4GHz CPU with an 8 GB DDR3 1800 MHz RAM.

4
https://www.cs.ucr.edu/∼eamonn/time_series_data/.

5 We used the py-ts library https://pyts.readthedocs.io/ to implement bf, the tslearn library https://tslearn.readthedocs.io to implement opt, and the scikit-learn library https://scikit-learn.org/stable/ to implement all the decision trees and the kNN.

6 For this particular experiment, parameters are set as follows: l = 30, h = 3, k = 100, and max_depth = 4.

7 In particular, this is also due to an optimization introduced by the library used for opt at prediction time.

8 1 indicates the best performer, and 4 indicates the worst performer. We exclude dtc and kNN as they do not work on shapelets.
