# Supplementary figures and images for: Matrix Profile-Based Interpretable Time Series Classifier
Source: Front Artif Intell. 2021 Oct 20;4:699448. doi: 10.3389/frai.2021.699448 (PMC8564499; doi:10.3389/frai.2021.699448)

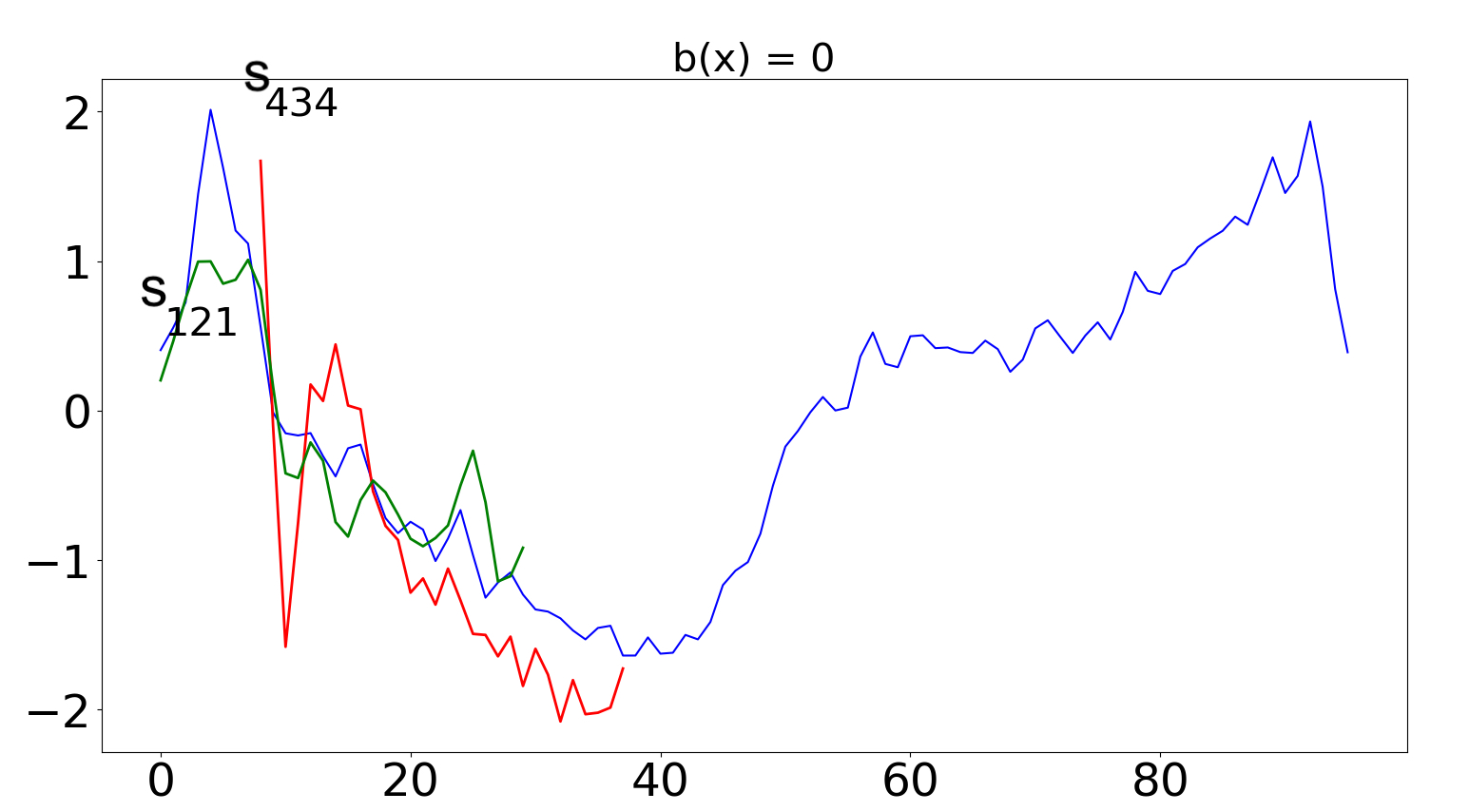

Supplement: Supplementary file 1 [file Image1.JPEG]

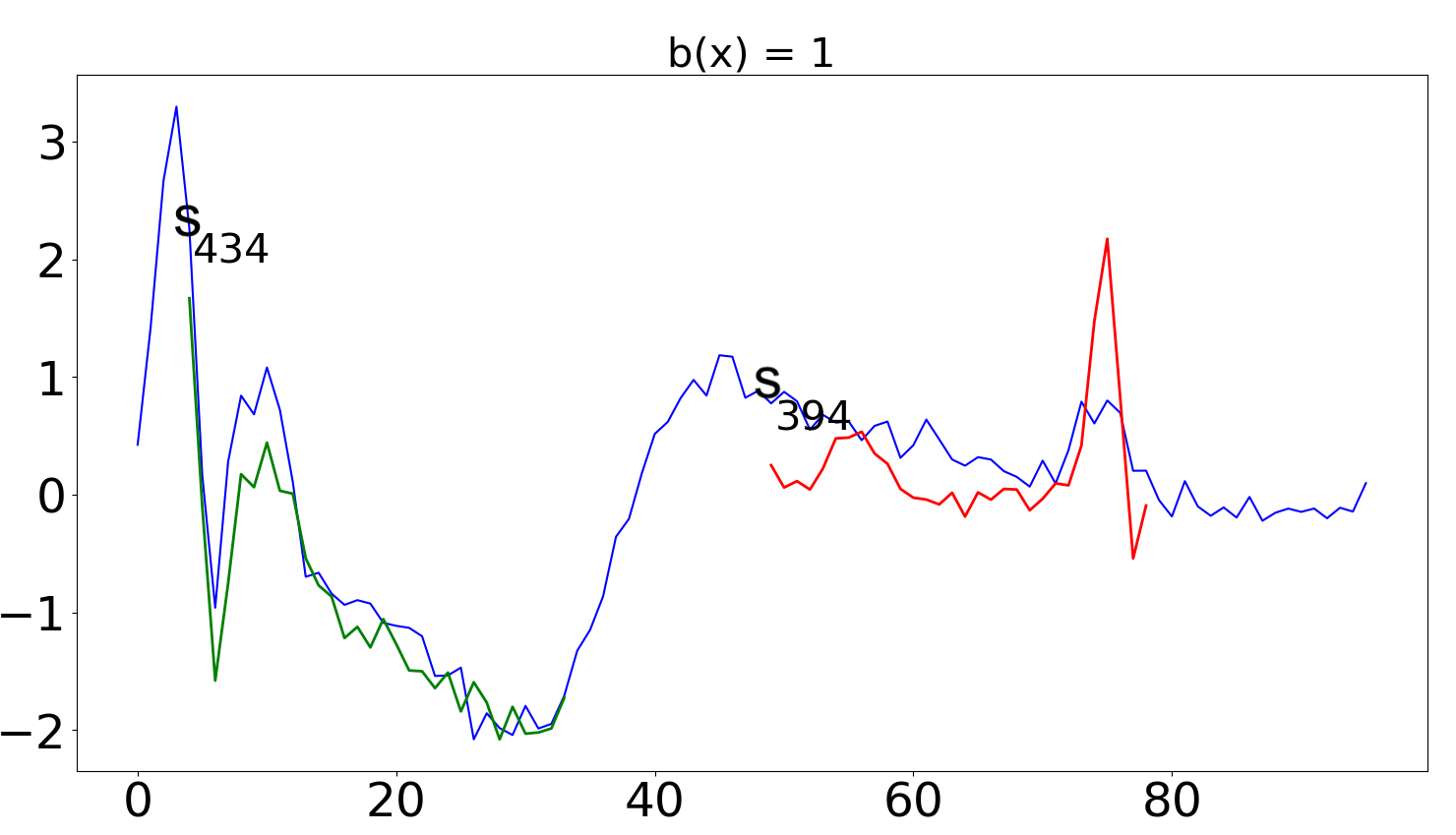

Supplement: Supplementary file 2 [file Image2.JPEG]
